# *Chlorella vulgaris* for Domestic Wastewater Treatment: Bibliometric Trends and Experimental Evaluation in Synthetic Effluent

**DOI:** 10.1002/wer.70276

**Published:** 2026-01-09

**Authors:** Letícia B. U. Melo, Bruna B. Borrego, Louise H. Gracioso, Marcos V. P. B. Campos, José J. Barrera‐Alba, Elen A. Perpetuo

**Affiliations:** ^1^ Institute of Marine Sciences (IMar‐UNIFESP), The Postgraduate Interdisciplinary Program in Marine Science and Technology (PPG‐ICTMar) Federal University of São Paulo Santos São Paulo Brazil; ^2^ Bio4Tec Lab, Environmental Research and Education Center University of São Paulo, CEPEMA‐POLI‐USP Cubatão Brazil; ^3^ The Interunits Postgraduate Program in Biotechnology University of São Paulo, PPIB‐USP São Paulo Brazil; ^4^ School of Arts, Science, and Humanities of University of São Paulo University of São Paulo, EACH‐USP São Paulo Brazil

**Keywords:** bibliometrics, circular economy, domestic effluent, microalgae, phycoremediation

## Abstract

Population growth has intensified domestic effluent generation, created environmental risks when inadequately treated. The microalga *Chlorella vulgaris* shows strong potential for wastewater remediation. This study combined bibliometric analysis and experimental validation to assess its application in synthetic domestic effluent treatment. Two strains, SL2C (mangrove origin) and BMAK D1 (freshwater origin), were cultivated in synthetic effluent at concentrations of 25%–100% (diluted in WC medium) for 14 days. Optimal growth occurred at 50% for SL2C, which achieved the highest specific growth rate (0.353 ± 0.003 d^−1^), and for BMAK D1, optimal growth occurred at 75% (0.262 ± 0.005 d^−1^). Effluent remediation was evaluated by monitoring ammoniacal nitrogen, phosphate, and chemical oxygen demand (COD). Nitrogen removal exceeded 80% across treatments, phosphate removal averaged ≈65%, and SL2C demonstrated greater COD reduction (66.9%) than BMAK D1 (54.9%). These results demonstrate the biotechnological value of *C. vulgaris* for wastewater treatment and its relevance to circular bioeconomy strategies, contributing to Sustainable Development Goal 6 (Clean Water and Sanitation).

## Summary

-
*Chlorella vulgaris* is a promising tool for domestic wastewater phycoremediation.
- Bibliometric analysis revealed research gaps and guided the experimental design.
-
*C. vulgaris* demonstrates favorable growth in high concentrations of synthetic wastewater.
- Mangrove strain exhibited rapid growth and high COD removal, reflecting possible metabolic adaptations to stressful environments.
- Using *C. vulgaris* in wastewater treatment supports the Sustainable Development Goals.

## Graphical abstract

This study combines bibliometric analysis and experimental assays to evaluate *Chlorella vulgaris* strains for domestic wastewater treatment. Findings highlight the phycoremediation potential of *C. vulgaris* toward sustainable water management and circular bioeconomy.

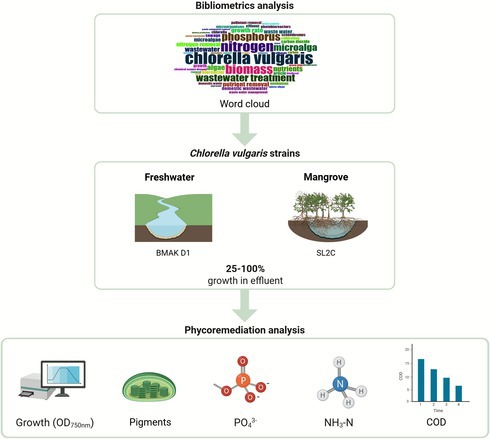

## 1 Introduction

The accelerated global population growth and ongoing industrialization have significantly contributed to worsening environmental pollution, driven by the unchecked emission of greenhouse gases (GHGs) and improper disposal of effluents (Leong and Chang 2023). These environmental impacts have a severe impact on ecosystems and human health, necessitating effective and sustainable solutions to mitigate such damage. Water quality degradation is another urgent concern: according to a 2017 UNESCO report, more than 2 million tons of wastewater and sewage are discharged daily into global water bodies (UNESCO 2017).

In response to these concerns, the United Nations (UN) launched the 2030 Agenda for Sustainable Development in 2015, a global action plan to eradicate poverty, protect the environment, and ensure quality of life for future generations. The agenda comprises 17 Sustainable Development Goals (SDGs) endorsed by all UN member states. These goals promote the rational use of natural resources and encourage the transition toward sustainable production and consumption models (Asadikia et al. 2021; Solarte‐Toro and Alzate 2021).

Biologically based technologies have emerged as promising tools for achieving the SDGs in this context. Among them, the use of photosynthesis via microalgal biomass stands out as an efficient solution capable of producing energy and high value bioproducts, ensuring food security, reducing reliance on fossil fuels, and most importantly, mitigating climate change (Catone et al. 2021; Rumin et al. 2020). Microalgae, unicellular organisms that can be prokaryotic (cyanobacteria) or eukaryotic (such as green algae), exhibit a CO_2_ fixation rate higher than that of terrestrial plants (Khan et al. 2018; Usui and Ikenouchi 1997).

Moreover, these microorganisms are remarkably adaptable to extreme environments, thriving across a wide range of temperatures, salinities, pH levels, and light intensities. They can grow in a wide variety of conditions, potentially replacing potable water with various wastewater (Khoo et al. 2021). These include domestic wastewater (Tran et al. 2021), effluents from tilapia culture systems (Bhuyar et al. 2021), and sugarcane processing wastewater (Silva et al. 2024). Moreover, *Chlorella vulgaris* can exist either as a free‐living organism or in symbiotic association with other microorganisms (Barsanti et al. 2008).

In this scenario, the bioremediation potential of microalgae is particularly relevant. Since the 1950s, studies have demonstrated their effectiveness in removing toxic compounds, including heavy metals, nitrogen, phosphorus, organic compounds, and carbon dioxide (Ali et al. 2023). Bioremediation may occur directly through nutrient uptake or indirectly via oxygen release, thereby enhancing the activity of aerobic decomposer microorganisms (Bala et al. 2022; Stiles et al. 2018).

Despite their potential, microalgae‐based systems for wastewater treatment still face economic and technical challenges. Consequently, research efforts have focused on optimizing and enabling more efficient and sustainable cultivation and biorefineries that can simultaneously meet environmental and economic goals (Leong and Chang 2023; Wang, Mukhambet, et al. 2022). In this context, the microalga *C. vulgaris* stands out as a promising candidate due to its adaptability to different environmental conditions, high nutrient uptake capacity, and potential for biomass valorization (Brandão et al. 2023; Mendes et al. 2024). These characteristics position it as a viable option for addressing current challenges in microalgae‐based wastewater treatment systems.

To identify prevailing scientific directions and areas requiring further investigation, statistical approaches such as bibliometric analysis have proven effective (Brandão et al. 2023). This method offers valuable insights and strategic direction for generating scientific data on emerging topics (Eregie et al. 2025). Therefore, this study initially aimed to conduct a bibliometric analysis to identify scientific trends, research gaps, and potential applications of *C. vulgaris* in the treatment of domestic wastewater. Based on these insights, the study evaluates the cell growth and phycoremediation potential of two *C. vulgaris* strains, isolated from distinct environments, cultivated under autotrophic and mixotrophic conditions using a synthetic domestic effluent. By analyzing nutrient removal efficiency, this research seeks to contribute to the advancement of sustainable wastewater treatment technologies in alignment with the SDGs.

## 2 Material and Methods

### 2.1 Data Acquisition and Bibliometric Analysis

The bibliographic search was conducted similar to that described by Melo et al. (2022). A literature search was conducted on June 25, 2025, using the Scopus and Web of Science Core Collection databases with the search terms “nutrient removal” AND “domestic wastewater” AND “ *Chlorella vulgaris* ”, restricted to the fields of title, abstract, and author keywords. The search covered the period from 2000 to 2025 and was limited to original research articles published in English, excluding reviews, conference papers, editorials, and technical notes.

A total of 83 publications were initially retrieved. These were subjected to a manual screening based on thematic relevance, resulting in the final selection of 41 articles for bibliometric analysis. The analysis was conducted using RStudio (version 2023.12.1) with the support of the Bibliometrix package, a widely used tool for mapping and visualizing bibliographic data.

### 2.2 Microalgae Strains

Strains of the microalga *C. vulgaris* , originating from two distinct locations, were kindly provided by institutions affiliated with the University of São Paulo (USP). The freshwater strain BMAK D1 was isolated from stream samples collected on the USP campus (São Paulo, Brazil) and was supplied by the Aidar & Kutner Marine Microorganism Bank (BMA&K). In turn, the strain SL2C was isolated from samples collected in mangrove areas in the municipality of Guarujá (23°54′48.9″S and 46°12′33.7″W, Brazil) and was provided by the Bio4Tec Lab Culture Collection (CEPEMA POLI USP).

### 2.3 Culture Medium

The selected microalgae were cultivated in two different media. The first one was WC medium (Wright's cryptophyte), a standard synthetic culture medium for microalgae, prepared according to the method described by Menezes et al. (2016). The second medium was a synthetic domestic effluent formulated to achieve a final COD of 500 mg O_2_ L^−1^. This effluent contained the following composition (per L^−1^): soy extract (312 mg), NaHCO_3_ (200 mg), sucrose (70 mg), starch (114 mg), NaCl (1.25 mg), MgCl_2_·6H_2_O (0.035 mg), CaCl_2_·2H_2_O (0.023 mg), KH_2_PO_4_ (0.132 mg), and commercial soy oil (0.05 mL) (adapted from Souza and Foresti 2013).

### 2.4 Microalgae Cultivation Conditions

The growth of *C. vulgaris* strains BMAK D1 and SL2C was evaluated in synthetic domestic effluent at concentrations of 25%, 50%, 75%, and 100%, which were obtained by diluting raw effluent with WC medium. These treatments were compared with a control using WC medium, all adjusted to pH 8. Cultures were maintained in 500 mL Erlenmeyer flasks containing 400 mL of working volume, under constant magnetic stirring, at a temperature of 27°C ± 3°C, and continuously illuminated by LED lamps at approximately 35 μmol m^−2^ s^−1^. The cultivation period for all conditions was 14 days.

### 2.5 Cellular Growth

Microalgae growth was monitored daily by measuring the optical density (OD) at 750 nm using a UV–Vis spectrophotometer (Shimadzu UV‐2450). These measurements were used to calculate the specific growth rate (μmax) and the generation time (GT), as described by Silva et al. (2024).

Photosynthetic pigment production (chlorophyll *a*, chlorophyll *b*, and carotenoids) was also evaluated. The samples were collected on the last day of cultivation and centrifuged at 8000 rpm for 5 min to separate the biomass. The pellet was resuspended in 0.5 mL of 80% (v v^−1^) acetone and homogenized by vortexing for 5 min. An additional 1.5 mL of 80% (v v^−1^) acetone was then added. The samples were protected from light, and incubated at −20°C for 30 min, followed by centrifugation at 8000 rpm for 10 min. The supernatant was analyzed in a UV–Vis spectrophotometer (Shimadzu UV‐2450). The absorbance was measured at 663.2 nm (chlorophyll *a*), 646.8 nm (chlorophyll *b*), and 470 nm (total carotenoids). Pigment concentrations were calculated using the following equations, as described by Lichtenthaler (1987) (Equations (1), (3)).

(1) $$\text{Chlorophyll}\;a\;\left(\mathrm{Ca}\right)=12.25\times A_{663.2\;\mathrm{nm}}-2.79\times A_{646.8\;\mathrm{nm}}$$

(2) $$\text{Chlorophyll}\;b\;\left(\mathrm{Cb}\right)=21.50\times A_{646.8\;\mathrm{nm}}-3.96\times A_{663.2\;\mathrm{nm}}$$

(3) $$\text{Total carotenoids}=\frac{1000\times A_{470\;\mathrm{nm}}-1.82\times \mathrm{Ca}-85.02\times \mathrm{Cb}}{198}$$

### 2.6 Effluent Treatment

Aliquots were collected at the end of cultivation and centrifuged at 8000 rpm for 10 min. The supernatants were used to assess the removal efficiency of compounds.

Ammoniacal nitrogen was quantified using the adapted indophenol blue method (Standard Methods for the Examination of Water and Wastewater 2025). For the reaction, 200 μL of phenol in ethanol (5% w v^−1^), 200 μL of sodium nitroprusside (0.5% w v^−1^), and 500 μL of oxidizing solution (160 g L^−1^ trisodium citrate, 8 g L^−1^ sodium hydroxide, and 1% sodium hypochlorite) were added to 500 μL of the supernatant. The mixture was incubated at room temperature under low‐light conditions for 1 h. A calibration curve was prepared using ammonium chloride, and absorbance was measured at 640 nm using a microplate reader (Biotek Synergy HTX).

Phosphate concentration was determined using the Spectroquant Phosphate Test Kit (Merck, Germany), following the manufacturer's instructions. COD was measured using the Spectroquant COD Cell Test Kit (Merck, Germany), according to the manufacturer's protocol.

### 2.7 Statistical Analysis

All the experiments and analyses were conducted in triplicate. Statistical differences between strains and treatments were compared using ANOVA and the Tukey test with Statistica Software (version 14.0).

## 3 Results and Discussion

### 3.1 Bibliometric Analysis

**FIGURE 1:**
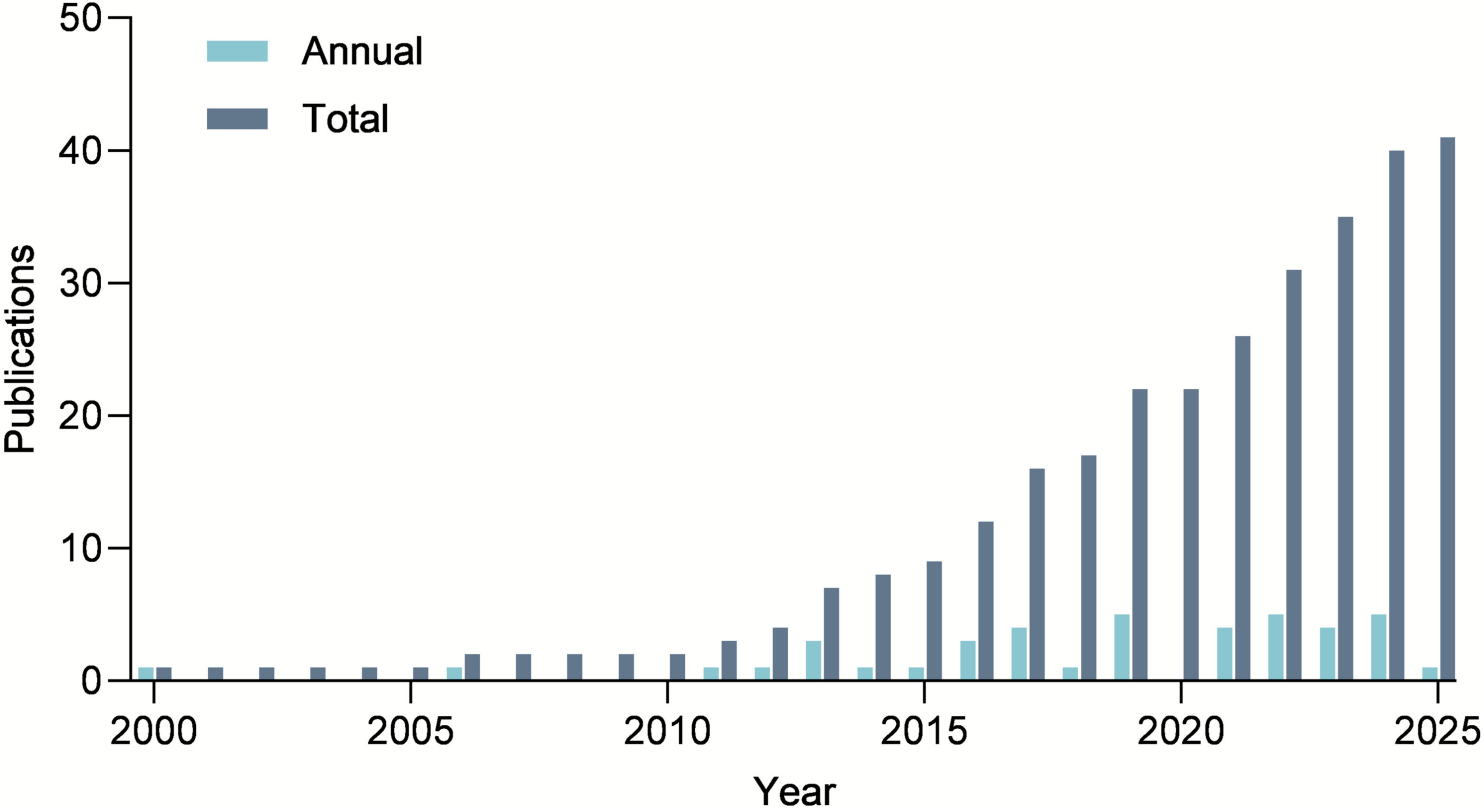
Annual and total number of scientific publications addressing *Chlorella vulgaris* in domestic wastewater treatment from 2000 to 2025.

The bibliometric analysis aimed to identify the main trends, gaps, and scientific patterns related to the application of *C. vulgaris* in domestic wastewater treatment. Publications prior to the year 2000 were excluded because of low output (only two publications). Figure 1 illustrates the temporal evolution of publications on the topic, showing a more pronounced increase from 2015 onward, which highlights the growing scientific interest in using *C. vulgaris* for wastewater treatment. Scientific output remained stable over the analyzed period, except in 2020, likely due to the redirection of biological research efforts toward studies on the novel coronavirus during the COVID‐19 pandemic (Whitaker et al. 2025). Despite this upward trend, the absolute number of studies remains relatively low, indicating a promising and expanding research area. In this context, the bibliometric analysis helps to guide the main research themes related to domestic wastewater treatment, allowing the identification of aspects that remain underexplored in the literature (Zhao, Peng, and Ma 2025).

**FIGURE 2:**
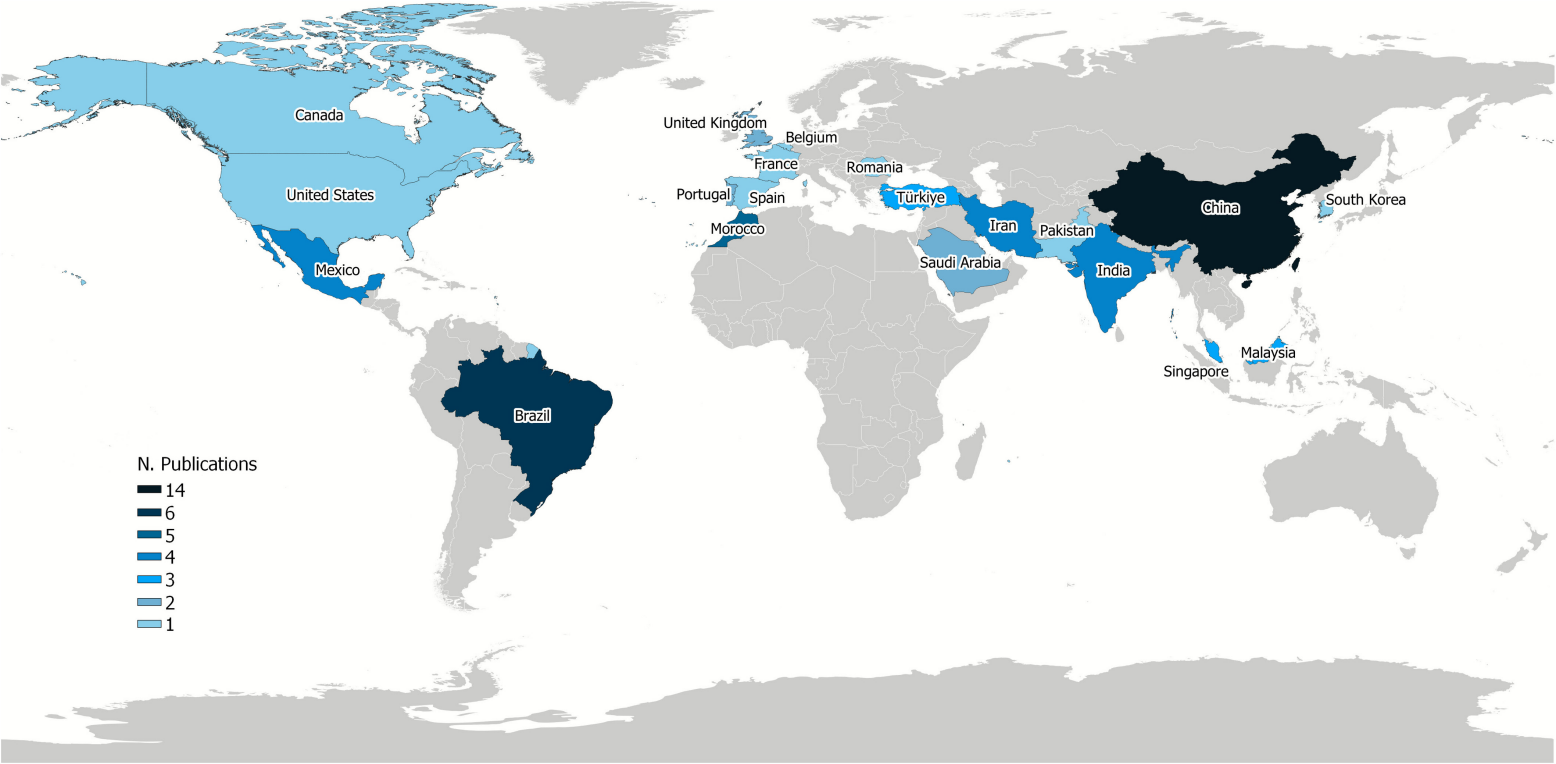
Scientific production on the treatment of domestic effluent by *Chlorella vulgaris* by country.

Figure 2 shows that the geographical distribution of these publications is mainly concentrated in the Americas, Asia, and Europe, with notable contributions from China (14), Brazil (6), and Morocco (5). This indicates a growing global interest in the topic, particularly in countries facing significant demands for wastewater treatment.

China stands out as the leading country in scientific production on this topic, a position driven by its rapid urbanization over recent decades, which has intensified both water consumption and water resource pollution (Lin et al. 2025). In the early 2020s, domestic wastewater generation in rural areas reached approximately 34 billion cubic meters, of which only 31% underwent adequate treatment (Zhao, Yang, et al. 2025). The discharge of untreated wastewater into stormwater drainage systems remains a common practice, which can overload urban treatment facilities and lead to significant environmental impacts (Yin et al. 2025).

Brazil also emerges as a notable contributor to the scientific literature on this subject, underscoring its potential for active participation in advancing the field. It is estimated that approximately 45% of domestic wastewater generated in Brazil does not receive proper treatment (Dantas et al. 2021). This situation is exacerbated by the country's continental dimensions, which contribute to marked regional disparities in access to sanitation services. While the Southeast and Central‐West regions treat over 50% of their generated domestic wastewater, the North and Northeast regions exhibit substantially lower rates, with only 22% to 33% of collected wastewater undergoing any form of treatment (Ferreira et al. 2021).

**FIGURE 3:**
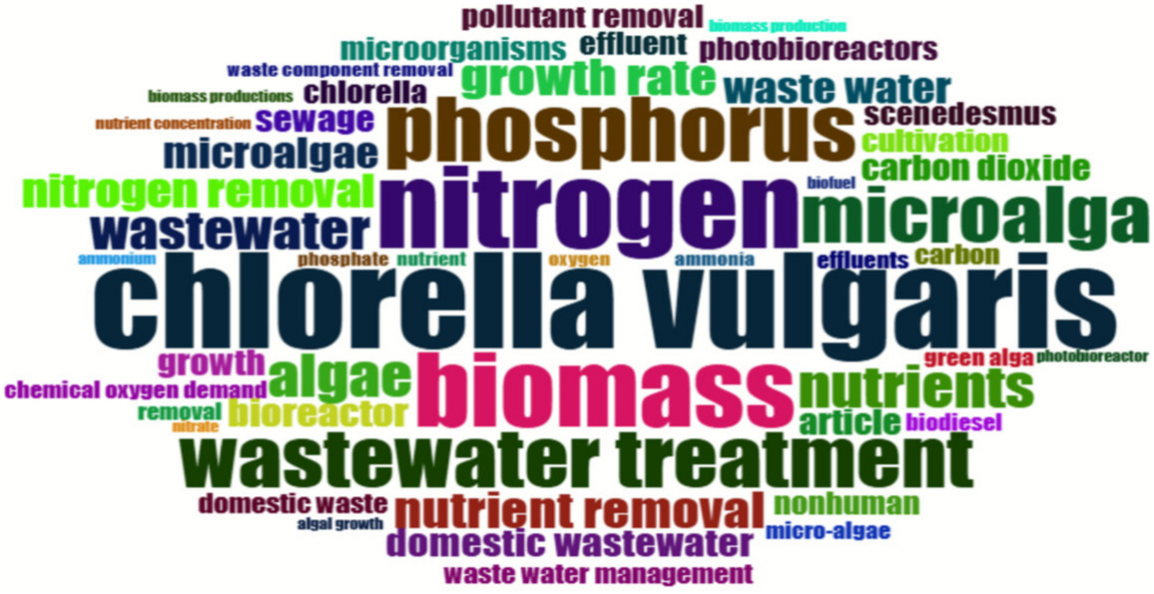
Word cloud representing the 50 most frequently used keywords in the selected publications. The size of each word is proportional to its frequency of occurrence.

The bibliometric analysis also revealed that the most frequently occurring terms in publications within this field include “ *Chlorella vulgaris* ”, “wastewater treatment”, “biomass”, “nitrogen”, “phosphorus”, and “nutrient removal” (Figure 3). These findings suggest that the current literature primarily focuses on the efficiency of *C. vulgaris* in removing nitrogen and phosphorus compounds, particularly from wastewater. Additionally, the term “domestic wastewater” appears with less prominence compared with “wastewater treatment”, suggesting that there is limited emphasis on using domestic effluent specifically for *C. vulgaris* cultivation. This highlights a research gap and opportunity for further exploration in systems targeting the treatment of domestic wastewater.

The recurrence of terms such as “nitrogen”, “phosphorus”, “chemical oxygen demand”, and “growth rate” reinforces the importance of evaluating the performance of *C. vulgaris* strains in terms of both nutrient removal efficiency and cell growth when cultivated in domestic wastewater as a culture medium.

The frequent occurrence of terms such as “biomass”, “biodiesel”, and “biofuels” suggests that *C. vulgaris* biomass cultivated in wastewater may hold significant biotechnological potential to produce bio‐based products for the energy industry. This highlights its strategic application as a sustainable alternative for waste valorization and environmental impact mitigation (Santos et al. 2025). This approach aligns with the principles of the circular bioeconomy, as it integrates wastewater treatment with the generation of high value bioproducts (Zabochnicka et al. 2022). In this context, Su et al. (2024) utilized the microalga *Tetradesmus dimorphus* GEEL‐06 for treating wastewater derived from domestic and pharmaceutical sources, simultaneously evaluating lipid production and fatty acid profiles, with a focus on biodiesel feasibility. Moreover, Lam et al. (2017) reported that low concentrations of domestic wastewater favor lipid accumulation, whereas higher concentrations can induce microalgal degradation. Nonetheless, biodiesel remains the most extensively studied bioproduct in the context of domestic wastewater treatment, underscoring the biotechnological potential of microalgal biomass (Brandão et al. 2023).

In summary, the application of *C. vulgaris* in treating domestic wastewater remains a relatively underexplored field, revealing promising opportunities for further research and scientific contributions. The prominent position of Brazil among the leading countries in scientific output on this topic highlights the importance of studies focused on the use of locally isolated strains. In this context, the comparison of different *C. vulgaris* strains isolated in Brazil, cultivated under controlled conditions in synthetic domestic wastewater, emerge as a promising strategy.

Therefore, based on the findings of the bibliometric analysis, the strategic objective of this study was to evaluate the cell growth performance of the selected strains and their efficiency in removing key nutrients, thereby contributing to the advancement of technical and scientific knowledge in this field.

### 3.2 Microalgae Growth

This study characterized the growth of two *C. vulgaris* strains in synthetic domestic effluent. One strain was obtained from a microalgae culture collection, while the other was isolated from a mangrove area located near the largest port in Latin America, in Santos (SP), a region subject to intense anthropogenic pressure (Borrego, Gracioso, et al. 2023). This isolation site was selected based on the premise that microorganisms adapted to adverse environmental conditions tend to exhibit greater resilience and a higher potential for growth in challenging media, such as wastewater (Yu et al. 2024). It is also noteworthy that literature lacks studies exploring the biotechnological potential of microalgae from such environments (Borrego, Melo, et al. 2023).

**FIGURE 4:**
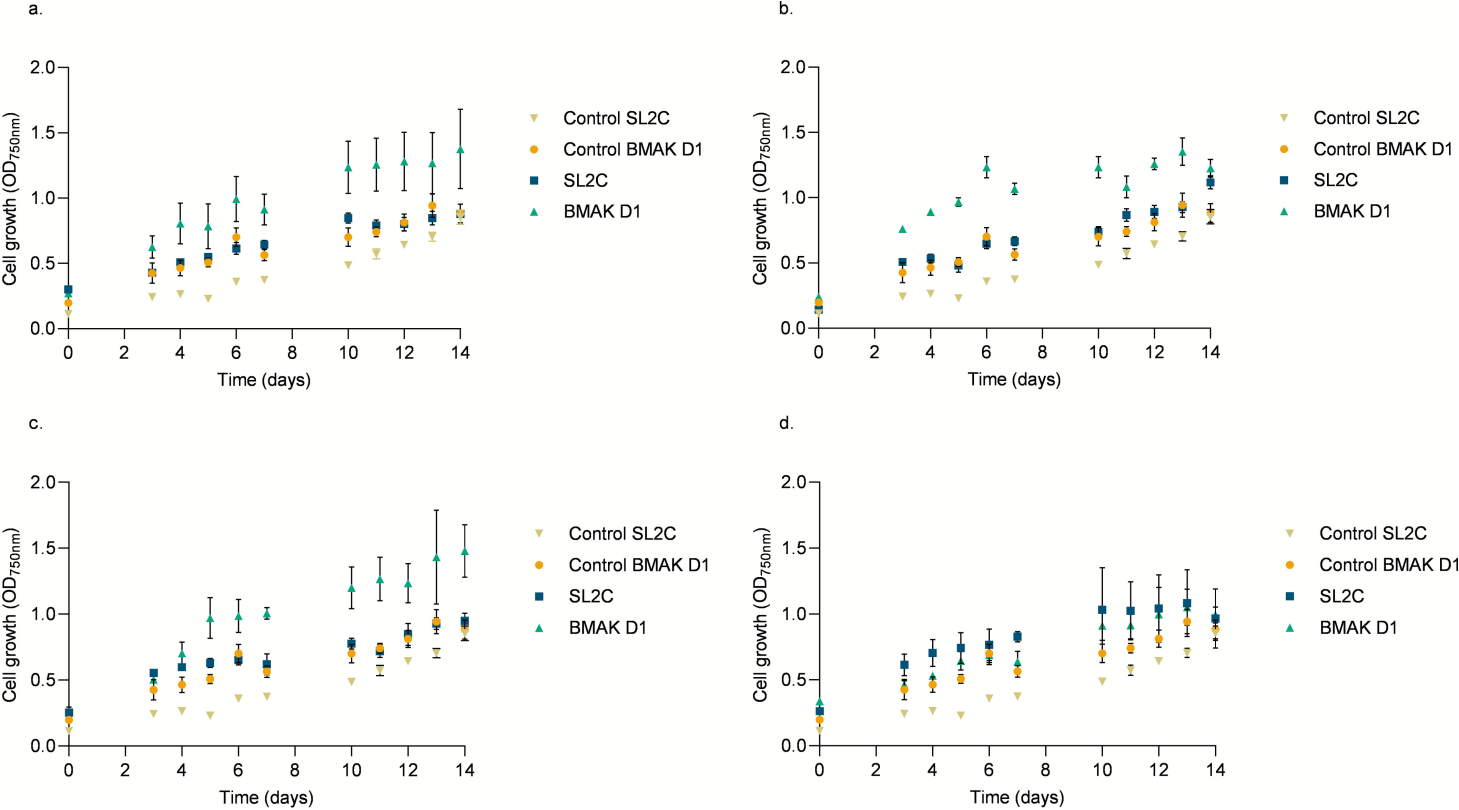
Cell growth by *Chlorella vulgaris* strains SL2C and BMAK D1 in different concentrations of effluent: 25% (a), 50% (b), 75% (c), and 100% (d).

In this study, both *Chlorella* strains exhibited daily increases in cell density monitored by absorbance at 750 nm. Growth was more pronounced in cultures with higher concentrations of synthetic wastewater compared with the control grown in WC medium, as shown in Figure 4, which presents the growth profiles under different effluent dilutions, ranging from 0% (control) to 100%.

Adequate nutrient availability is a key factor in promoting microalgal growth and biomass accumulation (Lam and Lee 2012). A study conducted by Xin et al. (2010) using a microalga from the genus *Scenedesmus* demonstrated that increasing nitrogen and phosphorus concentrations led to higher cell densities, highlighting the essential role of these nutrients in microalgal physiology.

The synthetic effluent used in this study contained soy extract and KH_2_PO_4_ as sources of nitrogen and phosphorus, respectively. Considering that soy contains approximately 40% protein (Guan et al. 2021), with nitrogen accounting for about 16% of this protein content, we estimate that this effluent had 1.4 times more nitrogen and 19 times more phosphorus than the WC medium. These findings support the conclusion that the presence of these nutrients enhances microalgal growth.

The highest absorbance values were recorded at 50% (1.117 ± 0.052) and 75% (1.480 ± 0.198) effluent concentrations for strains SL2C and BMAK D1, respectively. A similar behavior was observed for μmax, with SL2C showing the highest μmax at 50% (0.353 ± 0.003 d^−1^) effluent and BMAK D1 at 75% (0.262 ± 0.005 d^−1^) (Table 1).

**TABLE 1:** Growth kinetics of *Chlorella vulgaris* SL2C and BMAK D1 strains in different effluent concentrations (25%, 50%, 75%, and 100%). Different letters (a, b, c, d) indicate significant differences according to factorial ANOVA followed by Tukey's post hoc test. Variables were analyzed independently. GT represents generation time.

Strain	Condition	μmax (d^−1^)	GT (d)
SL2C	Control	0.109 ± 0.001^d^	6.341 ± 0.086^d^
	25%	0.103 ± 0.004^d^	6.749 ± 0.287^d^
	50%	0.353 ± 0.003^a^	1.963 ± 0.019^a^
	75%	0.227 ± 0.015^b^	3.061 ± 0.203^a,b^
	100%	0.253 ± 0.044^b^	2.799 ± 0.538^a^
BMAK D1	Control	0.200 ± 0.007^b,c^	3.475 ± 0.126^a,b^
	25%	0.221 ± 0.039^b^	3.205 ± 0.614^a,b^
	50%	0.153 ± 0.022^c,d^	4.595 ± 0.706^b,c^
	75%	0.262 ± 0.005^b^	2.648 ± 0.050^a^
	100%	0.122 ± 0.024^d^	5.823 ± 1.225^c,d^

As expected, generation time is inversely proportional to the growth rate; higher μmax corresponds to shorter generation time. The SL2C strain, which was isolated from a mangrove area subject to substantial anthropogenic impact, exhibited the fastest generation time (1.963 ± 0.019 days). This suggests a growth strategy adapted to environmental stress, characterized by growth with greater resilience. These findings highlight the potential of microalgal strains isolated from contaminated environments for use in effluent‐based bioprocesses.

Based on the μmax values, the generation time was calculated for all tested conditions. Statistical analyses indicated that both medium concentration and microalgal species significantly influenced the μmax, revealing a mutual interaction between these factors. In other words, the μmax response depends not only on each factor but on the combination of species and nutrient or effluent concentration. In contrast, no statistically significant differences were observed between species in terms of generation time. However, the interaction between species and concentration had a significant effect on generation time, indicating that although the species alone did not differ for this parameter, their response to the cultivation environment varied when both factors were considered together. This suggests that optimizing cultivation conditions requires selecting the appropriate species according to the applied concentration.

**FIGURE 5:**
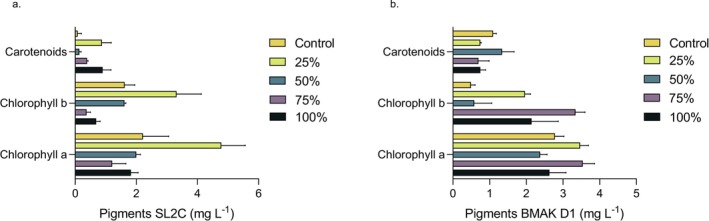
Pigments (chlorophyll a, chlorophyll b, and carotenoids) by *Chlorella vulgaris* strains SL2C (a) and BMAK D1 (b) in different effluent concentrations (25%, 50%, 75% and 100%).

Furthermore, the final pigments after 14 days were analyzed. Photosynthetic pigments, such as chlorophylls and carotenoids, play essential roles in phototrophic organisms (Ruan et al. 2025). Chlorophylls are directly involved in capturing and transferring light energy during photosynthesis (Li and Chen 2015), while carotenoids contribute to photoprotection and expand the spectrum of light absorption (Choudhury and Behera 2001). However, the synthesis of these pigments is strongly dependent on the availability of nitrogen. Nitrogen limitation impairs pigment biosynthesis, particularly chlorophyll, leading to decreased photosynthetic performance (Zhao et al. 2017). Figure 5 shows the final pigment concentrations in the cultures under different effluent dilution conditions, including the control.

Among all tested conditions, the lowest chlorophyll concentrations were observed in control treatments, with values not exceeding 1.3 mg L^−1^. This result may be explained by the reduced nitrogen concentration in the control medium, which, as previously mentioned, is approximately 1.4 times lower than that in the effluent‐based treatments.

In contrast, the highest chlorophyll concentrations were observed in the treatments at 75% effluent for the BMAK D1 strain and 25% effluent for the SL2C strain. For BMAK D1, chlorophyll *a* and *b* reached concentrations of 3.54 ± 0.322 mg L^−1^ and 3.340 ± 0.260 mg L^−1^, respectively. For SL2C, the maximum values were 4.781 ± 0.780 mg L^−1^ for chlorophyll *a* and 3.316 ± 0.811 mg L^−1^ for chlorophyll *b*.

In a study conducted by Fallahi et al. (2020), three microalgal species ( *C. vulgaris* , *Scenedesmus obliquus* , and *Nannochloropsis* sp.) were evaluated for growth, wastewater treatment, and pigment production using municipal effluent as the culture medium. The pigment concentrations reported were similarly low to those found in the present work for the same species, *C. vulgaris* , reaching 2.21 ± 0.3 mg L^−1^ for chlorophyll *a* and 1.71 ± 0.3 mg L^−1^ for chlorophyll *b*.

In another study using *C. vulgaris* , Pacheco et al. (2021) observed the highest total chlorophyll concentrations at 75% and 100% municipal effluent, reaching 0.86 and 2.38 mg L^−1^, respectively. Notably, the strain evaluated in our study exhibited even higher pigment accumulation under comparable conditions, with chlorophyll *a* concentration exceeding those reported in both studies. This suggests a strong physiological performance of our isolate, likely reflecting its adaptation to nutrient‐rich and potentially stressful environments.

It is worth noting that chlorophyll production in our study is relatively low when compared with other studies involving the same genus (*Chlorella*), such as those reported by Gupta et al. (2016), Bulynina et al. (2023), and Ruan et al. (2025). However, it is essential to note that the composition of the effluents used in each study can vary significantly, particularly in nitrogen concentration, which may positively impact pigment biosynthesis. Furthermore, a study by Lalucat et al. (1984) demonstrated that the presence of an external organic carbon source can modulate the metabolism of photosynthetic pigments, further supporting the variability observed across different cultivation conditions.

Carotenoid concentrations did not exceed 1.3 mg L^−1^ for the BMAK D1 strain and 0.8 mg L^−1^ for the SL2C strain, indicating reduced levels. As previously mentioned, one of the main functions of carotenoids is photoprotection. In our study, the cultures were maintained under continuous illumination, which may have induced some degree of stress; however, the light intensity was relatively low. This likely limited the need for photoprotective mechanisms, resulting in reduced carotenoid production by both strains.

### 3.3 Effluent Treatment

Phycoremediation has emerged as a promising approach for wastewater treatment, offering advantages in terms of cost‐effectiveness and environmental sustainability (Pacheco et al. 2015). Microalgae have demonstrated strong potential for bioremediation, as they can assimilate key nutrients commonly present in wastewater, such as nitrate, ammonia, and phosphate, which are essential for their growth and metabolism (Kaloudas et al. 2021). In particular, *C. vulgaris* is frequently employed in such applications due to its ability to thrive under mixotrophic conditions (Cao et al. 2023), its proven efficiency in pollutant removal (Kusuma et al. 2024), and the production of biomass enriched with high‐value biomolecules (Shafiei‐Alavijeh et al. 2024).

In this study, synthetic effluent was used as a model medium to evaluate the growth potential of selected microalgae under controlled nutrient conditions. This approach enables a more reproducible assessment of the metabolic performance and adaptability of the strains, while minimizing the variability and complexity typically found in real effluents (Singh and Mishra 2021; Wang, Wang, et al. 2022). The use of synthetic effluents also provides a baseline for anticipating the potential of these strains for application in real wastewater environments, where gradual adaptation strategies could be employed to increase their resilience and efficiency in bioremediation processes (Orantes‐Calleja et al. 2022).

**FIGURE 6:**
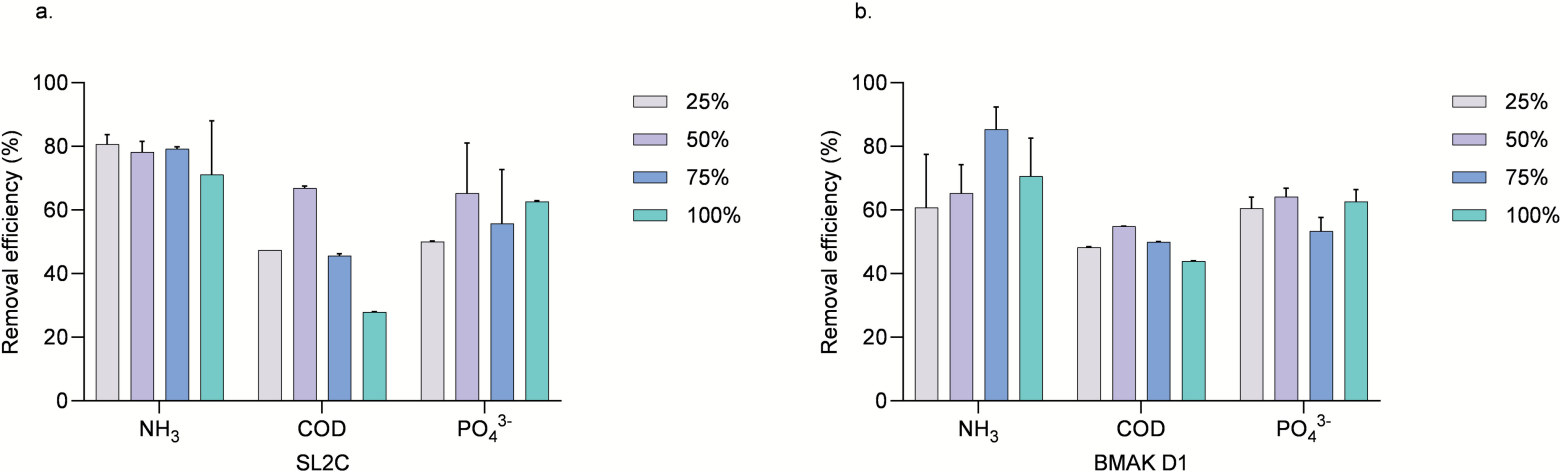
Removal efficiency (%) of ammoniacal nitrogen, COD, and phosphate at different effluent concentrations (25%, 50%, 75%, and 100%) by *Chlorella vulgaris* strains SL2C (a) and BMAK D1 (b). Different letters (a, b, c, d) indicate statistically significant differences according to one‐way ANOVA followed by Tukey's post hoc test. Variables were analyzed independently. Bars that do not share the same letter differ significantly (*p* < 0.05). n.s. represents nonsignificant differences between groups.

In this context, the removal efficiency of ammoniacal nitrogen (NH_3_—N), phosphate (PO_4_^3−^), and COD by the two *C. vulgaris* strains was evaluated (Figure 6). Regarding ammoniacal nitrogen, both strains demonstrated high removal efficiency, regardless of the effluent concentration.

The initial concentration and the concentration after effluent treatment were also verified (Table 2). Notably, the SL2C strain achieved removal rates consistently above 70% under all tested conditions. This result aligns with previous reports highlighting the ability of *C. vulgaris* to assimilate ammoniacal nitrogen from wastewater efficiently (Göncü et al. 2025), due to its role as an essential source of nitrogen in microalgae metabolism, primarily in the form of ammonium (Salbitani and Carfagna 2021). Effective ammoniacal nitrogen removal is particularly relevant, as excess nitrogen in wastewater contributes to several environmental problems, including eutrophication and the subsequent reduction of oxygen and toxicity in aquatic ecosystems (Akinnawo 2023).

**TABLE 2:** Nutrient concentrations before and after phycoremediation using *Chlorella vulgaris* strains SL2C and BMAK D1 cultured in synthetic effluent at 25%, 50%, 75%, and 100% concentrations.

		N—NH_3_ (mg L^−1^)		COD (mg O_2_ L^−1^)		PO_4_^3−^ (mg L^−1^)	
		Initial	Final	Initial	Final	Initial	Final
SL2C	25%	1.28	0.25 ± 0.02	173.30	91.17 ± 0.01	8.89	4.44 ± 0.04
	50%	2.36	0.51 ± 0.08	278.60	92.18 ± 0.67	11.82	4.10 ± 1.87
	75%	3.60	0.75 ± 0.02	378.90	205.99 ± 0.63	14.10	6.23 ± 1.38
	100%	4.87	1.41 ± 0.82	537.20	368.84 ± 0.01	15.55	5.81 ± 0.06
BMAK D1	25%	1.28	0.50 ± 0.21	173.30	89.68 ± 0.18	8.89	3.50 ± 0.31
	50%	2.36	0.82 ± 0.30	278.60	125.70 ± 0.11	11.82	4.23 ± 0.41
	75%	3.60	0.53 ± 0.25	378.90	189.66 ± 0.16	14.10	6.57 ± 0.61
	100%	4.87	1.43 ± 0.59	537.20	301.03 ± 0.11	15.55	5.81 ± 0.60

In terms of COD, the highest removal was observed for the SL2C strain at 66.91%, using a 50% effluent concentration. However, this strain showed a marked decrease in COD removal efficiency at higher effluent concentrations, dropping to 28% at 100% effluent. Conversely, the BMAK D1 strain demonstrated more consistent COD removal performance across all concentrations, with efficiencies ranging from 43.96% to 54.88%. These results align with previous studies that report the capacity of *C. vulgaris* to reduce COD levels of different wastewater (Soto et al. 2021; Zhu et al. 2022), regardless of the metabolic cultivation conditions (Ge et al. 2018). COD removal is crucial, as it reflects the reduction of both biodegradable and nonbiodegradable pollutants, thereby improving water quality (Li et al. 2020).

Phosphate removal by both strains remained moderate and relatively stable across all effluent concentrations tested. Previous studies have similarly demonstrated the potential of *C. vulgaris* to reduce phosphate content in wastewater, mitigating the risk of eutrophication and promoting sustainable nutrient recycling (Santos and Pires 2018). For instance, Vazirzadeh et al. (2022) reported approximately 95% phosphate removal in simulated agricultural runoff. Ajala and Alexander (2020) observed efficiencies ranging from 84% to 94% in secondary municipal wastewater. Additionally, Kong et al. (2021) demonstrated phosphate removal ranging from 38% to 61% in artificial wastewater under atmospheric CO_2_ supplementation. Hiltunen et al. (2026) evaluated the phosphorus removal efficiency of *C. vulgaris* even in cultures supplemented with additional phosphorus, thereby altering the N/P ratio, and found that under all conditions, the microalga absorbed nearly all available phosphorus.

Overall, the 50% effluent concentration appeared particularly promising from a biotechnological standpoint. Under these conditions, the highest COD removal was achieved by both strains, with SL2C showing a significantly greater removal capacity (*p* < 0.05). Mangrove‐derived microalgae exhibit unique physiological and biochemical properties, providing potential for various biotechnological applications. Moreover, the Baixada Santista mangrove, which experiences intense anthropogenic impacts and is among the most polluted coastal areas in Brazil, may harbor strains with high adaptability to adverse and stressful environmental conditions (Borrego et al. 2025). Additionally, the removal of ammoniacal nitrogen and phosphate remained satisfactory. This condition not only supports effective pollutant removal and cellular growth but also reduces the need for potable water input, thereby enhancing the sustainability and economic feasibility of microalgal cultivation systems for biomass production (Chaudry 2021). In this context, Kumaran et al. (2023) investigated lipid production by *C. vulgaris* in palm oil mill effluent (POME), aiming to enhance the value of the resulting microalgal biomass. These findings reinforce the importance of strain selection based on environmental origin. The SL2C strain, adapted to a highly impacted habitat, likely possesses metabolic versatility and stress‐response mechanisms that enhance its ability to degrade complex organic compounds, ultimately resulting in higher COD removal efficiency.

These findings underscore the importance of incorporating microalgal technologies into sustainable wastewater management strategies, with direct implications for the achievement of several SDGs. The application of microalgae in treating domestic wastewater, coupled with the generation of bioproducts, exemplifies a circular bioeconomy approach (Olabi et al. 2023). This study supports the goals of SDG 6, which promotes access to clean water and sanitation (United Nations 2025), as the cultivation process led to effective remediation of synthetic domestic effluent, significantly reducing organic matter, nitrogen, and phosphorus, that are key contributors to water pollution (Chai et al. 2021).

Moreover, the microalgae's high CO_2_ fixation capacity, reported to be up to 10 times greater than that of terrestrial plants (Hachicha et al. 2022), aligns with the targets of SDG 13, which aims to mitigate climate change and its impacts. By removing excess nutrients from wastewater, this process also contributes to SDG 14, which focuses on conserving marine ecosystems by helping to prevent eutrophication in coastal and oceanic environments (Olabi et al. 2023).

Finally, the residual biomass generated presents a promising feedstock for biofuel production, particularly biodiesel through lipid extraction (Sati et al. 2019), thus aligning with SDG 7, which advocates for affordable and clean energy. This connection between wastewater treatment and energy generation is further supported by a bibliometric analysis (Figure 3), which highlights the growing interest in the scientific community for the valorization of microalgal biomass as a sustainable and value‐added solution.

## 4 Conclusion

The phycoremediation of domestic effluents has garnered increasing scientific interest, particularly in countries such as China, Brazil, and Morocco, where wastewater management remains a critical challenge. Within this context, *C. vulgaris* stands out as a promising candidate for sustainable treatment solutions. In this study, two strains of *C. vulgaris* , isolated from distinct environments, were evaluated for their ability to treat synthetic domestic effluent. Strain SL2C, isolated from a mangrove under strong anthropogenic influence, and strain BMAK D1, obtained from a culture collection, both demonstrated optimal performance at 50% effluent concentration diluted in WC medium. SL2C achieved higher COD removal and specific growth rate, likely reflecting its metabolic versatility and adaptation to stressful environments. These findings support the potential use of native microalgae in real wastewater treatment systems, contributing to clean water initiatives and sustainable biotechnological solutions. Thus, the integration of *C. vulgaris* into treatment systems offers a viable strategy for promoting environmental sustainability and public health. Future research should focus on evaluating *C. vulgaris* in real wastewater to elucidate its physiological responses under complex conditions and to assess its practical applicability as a sustainable and complementary stage in conventional wastewater treatment systems.

## Author Contributions

**Letícia B. U. Melo:** methodology, investigation, visualization, formal analysis, writing – original draft. **Bruna B. Borrego:** methodology, formal analysis, writing – original draft. **Louise H. Gracioso:** validation, writing – original draft. **Marcos V. P. B. Campos:** methodology, investigation, formal analysis, writing – review and editing. **José J. Barrera‐Alba:** conceptualization, supervision, writing – review and editing. **Elen A. Perpetuo:** conceptualization, resources, writing – review and editing, funding acquisition, supervision.

## Funding

This work was supported by Coordenação de Aperfeiçoamento de Pessoal de Nível Superior 001; Fundação de Amparo à Pesquisa do Estado de São Paulo 2021/04367‐2.

## Conflicts of Interest

The authors declare no conflicts of interest.

## Data Availability

The data that support the findings of this study are available from the corresponding author upon reasonable request.

## References

[wer70276-bib-0001] Ajala, S. O. , and M. L. Alexander . 2020. “Assessment of Chlorella vulgaris, Scenedesmus obliquus, and Oocystis minuta for Removal of Sulfate, Nitrate, and Phosphate in Wastewater.” International Journal of Energy and Environmental Engineering 11, no. 3: 311–326. 10.1007/s40095-019-00333-0.

[wer70276-bib-0002] Akinnawo, S. O. 2023. “Eutrophication: Causes, Consequences, Physical, Chemical and Biological Techniques for Mitigation Strategies.” Environmental Challenges 12: 100733. 10.1016/j.envc.2023.100733.

[wer70276-bib-0003] Ali, A. , Z. Khalid , A. Ahmed , and J. Ajarem . 2023. “Wastewater Treatment by Using Microalgae: Insights Into Fate, Transport, and Associated Challenges.” Chemosphere 338: 139501. 10.1016/j.chemosphere.2023.139501. 37453525

[wer70276-bib-0004] Asadikia, A. , A. Rajabifard , and M. Kalantari . 2021. “Systematic Prioritisation of SDGs: Machine Learning Approach.” World Development 140: 105269. 10.1016/j.worlddev.2020.105269.

[wer70276-bib-0005] Bala, S. , D. Garg , B. V. Thirumalesh , et al. 2022. “Recent Strategies for Bioremediation of Emerging Pollutants: A Review for a Green and Sustainable Environment.” Toxics 10, no. 8: 484. 10.3390/toxics10080484. 36006163 PMC9413587

[wer70276-bib-0006] Barsanti, L. , P. Coltelli , V. Evangelista , et al. 2008. “Oddities and Curiosities in the Algal World.” In Algal Toxins: Nature, Occurrence, Effect and Detection (Algal Toxins), 353–391. Springer Netherlands. 10.1007/978-1-4020-8480-5_17.

[wer70276-bib-0007] Bhuyar, P. , M. Trejo , N. Dussadee , Y. Unpaprom , R. Ramaraj , and K. Whangchai . 2021. “Microalgae Cultivation in Wastewater Effluent From Tilapia Culture Pond for Enhanced Bioethanol Production.” Water Science and Technology 84, no. 10–11: 2686–2694. 10.2166/wst.2021.194. 34850686

[wer70276-bib-0008] Borrego, B. B. , L. H. Gracioso , B. Karolski , et al. 2023. “Tributyltin Degrading Microbial Enzymes: A Promising Remediation Approach.” Marine Pollution Bulletin 189: 114725. 10.1016/j.marpolbul.2023.114725. 36805770

[wer70276-bib-0009] Borrego, B. B. , L. B. U. Melo , L. H. Gracioso , L. O. B. Cardoso , and E. A. Perpetuo . 2023. “Photoautotrophic Microorganisms From Mangroves: A Review of the Ecological Role and Bioproducts of Commercial Interest.” Biofuels, Bioproducts and Biorefining 17, no. 5: 1457–1477. 10.1002/bbb.2515.

[wer70276-bib-0010] Borrego, B. B. , F. L. Oliveira , L. B. U. Melo , et al. 2025. “Unveiling Metabolic Diversity Through Phylogenetic Analysis and Carbohydrate Composition of Microalgae Isolated From Mangroves in Brazil.” Algal Research 91: 104313. 10.1016/j.algal.2025.104313.

[wer70276-bib-0017] Brandão, B. C. S. , C. Y. B. Oliveira , E. P. Dos Santos , et al. 2023. “Microalgae‐Based Domestic Wastewater Treatment: A Review of Biological Aspects, Bioremediation Potential, and Biomass Production With Biotechnological High‐Value.” Environmental Monitoring and Assessment 195, no. 11: 1384. 10.1007/s10661-023-12031-w. 37889346

[wer70276-bib-0011] Bulynina, S. S. , E. E. Ziganshina , and A. M. Ziganshin . 2023. “Growth Efficiency of Chlorella sorokiniana in Synthetic Media and Unsterilized Domestic Wastewater.” Biotech 12, no. 3: 53. 10.3390/biotech12030053. 37606440 PMC10443301

[wer70276-bib-0012] Cao, Y. , S. Yang , J. Wang , et al. 2023. “Metabolomic Exploration of the Physiological Regulatory Mechanism of the Growth and Metabolism Characteristics of Chlorella vulgaris Under Photoautotrophic, Mixotrophic, and Heterotrophic Cultivation Conditions.” Biomass and Bioenergy 173: 106775. 10.1016/j.biombioe.2023.106775.

[wer70276-bib-0013] Catone, C. M. , M. Ripa , E. Geremia , and S. Ulgiati . 2021. “Bio‐Products From Algae‐Based Biorefinery on Wastewater: A Review.” Journal of Environmental Management 293: 112792. 10.1016/j.jenvman.2021.112792. 34058450

[wer70276-bib-0014] Chai, W. S. , W. G. Tan , H. S. Halimatul Munawaroh , V. K. Gupta , S.‐H. Ho , and P. L. Show . 2021. “Multifaceted Roles of Microalgae in the Application of Wastewater Biotreatment: A Review.” Environmental Pollution 269: 116236. 10.1016/j.envpol.2020.116236. 33333449

[wer70276-bib-0015] Chaudry, S. 2021. “Integrating Microalgae Cultivation With Wastewater Treatment: A Peek Into Economics.” Applied Biochemistry and Biotechnology 193, no. 10: 3395–3406. 10.1007/s12010-021-03612-x. 34196918

[wer70276-bib-0016] Choudhury, N. K. , and R. K. Behera . 2001. “Photoinhibition of Photosynthesis: Role of Carotenoids in Photoprotection of Chloroplast Constituents.” Photosynthetica 39, no. 4: 481–488. 10.1023/A:1015647708360.

[wer70276-bib-0051] Dantas, M. S. , G. R. Barroso , and S. C. Oliveira . 2021. “Performance of Sewage Treatment Plants and Impact of Effluent Discharge on Receiving Water Quality Within an Urbanized Area.” Environmental Monitoring and Assessment 193, no. 5: 289. 10.1007/s10661-021-09075-1. 33886032

[wer70276-bib-0018] Eregie, S. B. , I. A. Sanusi , and O. O. Ademola . 2025. “Current Status and Future Prospects of Microalgae‐Based Degradation of Spent Lubricant Oil Hydrocarbon Towards Environmental Sustainability: A Mini Review and Bibliometric Analysis.” Archives of Microbiology 207, no. 7: 149. 10.1007/s00203-025-04332-0. 40387936 PMC12089184

[wer70276-bib-0019] Fallahi, A. , N. Hajinajaf , O. Tavakoli , and M. H. Sarrafzadeh . 2020. “Cultivation of Mixed Microalgae Using Municipal Wastewater: Biomass Productivity, Nutrient Removal, and Biochemical Content.” Iranian Journal of Biotechnology 18, no. 4: 88–97. 10.30498/IJB.2020.2586. PMC8148641 34056025

[wer70276-bib-0020] Ferreira, M. M. , F. A. Fiore , A. Saron , and G. H. R. da Silva . 2021. “Systematic Review of the last 20 Years of Research on Decentralized Domestic Wastewater Treatment in Brazil: State of the Art and Potentials.” Water Science and Technology 84, no. 12: 3469–3488. 10.2166/wst.2021.487. 34928820

[wer70276-bib-0021] Ge, S. , S. Qiu , D. Tremblay , K. Viner , P. Champagne , and P. G. Jessop . 2018. “Centrate Wastewater Treatment With Chlorella vulgaris: Simultaneous Enhancement of Nutrient Removal, Biomass and Lipid Production.” Chemical Engineering Journal 342: 310–320. 10.1016/j.cej.2018.02.058.

[wer70276-bib-0022] Göncü, S. , B. Şimşek Uygun , and S. Atakan . 2025. “Nitrogen and Phosphorus Removal From Wastewater Using Chlorella vulgaris and Scenedesmus quadricauda Microalgae With a Batch Bioreactor.” International Journal of Environmental Science and Technology 22: 11877–11892. 10.1007/s13762-025-06380-x.

[wer70276-bib-0023] Guan, X. , X. Zhong , Y. Lu , et al. 2021. “Changes of Soybean Protein During Tofu Processing.” Food 10, no. 7: 1594. 10.3390/foods10071594. PMC8306988 34359464

[wer70276-bib-0024] Gupta, P. L. , H.‐J. Choi , R. R. Pawar , S. P. Jung , and S.‐M. Lee . 2016. “Enhanced Biomass Production Through Optimization of Carbon Source and Utilization of Wastewater as a Nutrient Source.” Journal of Environmental Management 184: 585–595. 10.1016/j.jenvman.2016.10.018. 27789093

[wer70276-bib-0025] Hachicha, R. , F. Elleuch , H. Ben Hlima , et al. 2022. “Biomolecules From Microalgae and Cyanobacteria: Applications and Market Survey.” Applied Sciences 12, no. 4: 1924. 10.3390/app12041924.

[wer70276-bib-0026] Hiltunen, M. , R. Kannangara , B. Nakandalage , et al. 2026. “Growth and Biomass Composition of Chlorella vulgaris Using Nutrient‐Rich Water and CO2 From a Recirculating Aquaculture System.” Aquaculture 610: 742956. 10.1016/j.aquaculture.2025.742956.

[wer70276-bib-0027] Kaloudas, D. , N. Pavlova , and R. Penchovsky . 2021. “Phycoremediation of Wastewater by Microalgae: A Review.” Environmental Chemistry Letters 19, no. 4: 2905–2920. 10.1007/s10311-021-01203-0.

[wer70276-bib-0028] Khan, M. I. , J. H. Shin , and J. D. Kim . 2018. “The Promising Future of Microalgae: Current Status, Challenges, and Optimization of a Sustainable and Renewable Industry for Biofuels, Feed, and Other Products.” Microbial Cell Factories 17, no. 1: 36. 10.1186/s12934-018-0879-x. 29506528 PMC5836383

[wer70276-bib-0029] Khoo, K. S. , W. Y. Chia , K. W. Chew , and P. L. Show . 2021. “Microalgal‐Bacterial Consortia as Future Prospect in Wastewater Bioremediation, Environmental Management and Bioenergy Production.” Indian Journal of Microbiology 61, no. 3: 262–269. 10.1007/s12088-021-00924-8. 34294991 PMC8263830

[wer70276-bib-0030] Kong, W. , J. Kong , J. Ma , et al. 2021. “Chlorella vulgaris Cultivation in Simulated Wastewater for the Biomass Production, Nutrients Removal and CO_2_ Fixation Simultaneously.” Journal of Environmental Management 284: 112070. 10.1016/j.jenvman.2021.112070. 33561760

[wer70276-bib-0031] Kumaran, M. , K. M. Palanisamy , P. Bhuyar , G. P. Maniam , M. H. Rahim , and N. Govindan . 2023. “Agriculture of Microalgae Chlorella vulgaris for Polyunsaturated Fatty Acids (PUFAs) Production Employing Palm Oil Mill Effluents (POME) for Future Food, Wastewater, and Energy Nexus.” Energy Nexus 9: 100169. 10.1016/j.nexus.2022.100169.

[wer70276-bib-0032] Kusuma, H. S. , N. Illiyanasafa , D. E. C. Jaya , H. Darmokoesoemo , and N. R. Putra . 2024. “Utilization of the Microalga Chlorella vulgaris for Mercury Bioremediation From Wastewater and Biomass Production.” Sustainable Chemistry and Pharmacy 37: 101346. 10.1016/j.scp.2023.101346.

[wer70276-bib-0033] Lalucat, J. , J. Imperial , and R. Parés . 1984. “Utilization of Light for the Assimilation of Organic Matter in *Chlorella* sp. VJ79.” Biotechnology and Bioengineering 26, no. 7: 677–681. 10.1002/bit.260260707. 18553430

[wer70276-bib-0034] Lam, M. K. , and K. T. Lee . 2012. “Potential of Using Organic Fertilizer to Cultivate Chlorella vulgaris for Biodiesel Production.” Applied Energy 94: 303–308. 10.1016/j.apenergy.2012.01.075.

[wer70276-bib-0035] Lam, M. K. , M. I. Yusoff , Y. Uemura , et al. 2017. “Cultivation of Chlorella vulgaris Using Nutrients Source From Domestic Wastewater for Biodiesel Production: Growth Condition and Kinetic Studies.” Renewable Energy 103: 197–207. 10.1016/j.renene.2016.11.032.

[wer70276-bib-0036] Leong, Y. K. , and J.‐S. Chang . 2023. “Waste Stream Valorization‐Based Low‐Carbon Bioeconomy Utilizing Algae as a Biorefinery Platform.” Renewable and Sustainable Energy Reviews 178: 113245. 10.1016/j.rser.2023.113245.

[wer70276-bib-0037] Li, L.‐H. , X.‐Y. Li , Y. Hong , M.‐R. Jiang , and S.‐L. Lu . 2020. “Use of Microalgae for the Treatment of Black and Odorous Water: Purification Effects and Optimization of Treatment Conditions.” Algal Research 47: 101851. 10.1016/j.algal.2020.101851.

[wer70276-bib-0038] Li, Y. , and M. Chen . 2015. “Novel chlorophylls and New Directions in Photosynthesis Research.” Functional Plant Biology 42, no. 6: 493–501. 10.1071/FP14350. 32480695

[wer70276-bib-0039] Lightenthaler, H. K. 1987. “Chlorophylls and Carotenoids: Pigments of Photosynthetic Biomembranes.” Methods in Enzymology 148: 350–382.

[wer70276-bib-0040] Lin, Z. , M. Li , P. Yan , J. Zhang , H. Xie , and H. Wu . 2025. “Constructed Wetlands for Wastewater Treatment and Reuse: Two Decades of Experience From China.” Environmental Research 279: 121781. 10.1016/j.envres.2025.121781. 40335010

[wer70276-bib-0041] Melo, J. M. , M. R. Ribeiro , T. S. Telles , H. F. Amaral , and D. S. Andrade . 2022. “Microalgae Cultivation in Wastewater From Agricultural Industries to Benefit Next Generation of Bioremediation: A Bibliometric Analysis.” Environmental Science and Pollution Research 29, no. 15: 22708–22720. 10.1007/s11356-021-17427-0. 34797540

[wer70276-bib-0042] Mendes, A. R. , M. P. Spínola , M. Lordelo , and J. A. M. Prates . 2024. “Advances in Bioprocess Engineering for Optimising Chlorella vulgaris Fermentation: Biotechnological Innovations and Applications.” Food 13, no. 24: 4154. 10.3390/foods13244154. PMC11675943 39767096

[wer70276-bib-0043] Menezes, R. S. , A. T. Soares , J. G. Marques Júnior , et al. 2016. “Culture Medium Influence on Growth, Fatty Acid, and Pigment Composition of Choricystis minor var. minor: A Suitable Microalga for Biodiesel Production.” Journal of Applied Phycology 28, no. 5: 2679–2686. 10.1007/s10811-016-0828-1.

[wer70276-bib-0044] Olabi, A. G. , N. Shehata , E. T. Sayed , et al. 2023. “Role of Microalgae in Achieving Sustainable Development Goals and Circular Economy.” Science of the Total Environment 854: 158689. 10.1016/j.scitotenv.2022.158689. 36108848

[wer70276-bib-0045] Orantes‐Calleja, P. D. , P. López de Paz , A. Rosales Quintero , C. A. Meza Avendaño , L. Verea , and K. E. Díaz Santiago . 2022. “Comparative Optimization of Macronutrient Removal from a Cyanobacterium and a Microalga Grown in Synthetic Wastewater for Potential Use as a Biodiesel Source.” Biofuels 13, no. 8: 1041–1053. 10.1080/17597269.2022.2079247.

[wer70276-bib-0046] Pacheco, D. , A. C. S. Rocha , A. Garcia , A. Bóia , L. Pereira , and T. Verdelhos . 2021. “Municipal Wastewater: A Sustainable Source for the Green Microalgae Chlorella vulgaris Biomass Production.” Applied Sciences 11, no. 5: 2207. 10.3390/app11052207.

[wer70276-bib-0047] Pacheco, M. M. , M. Hoeltz , M. S. A. Moraes , and R. C. S. Schneider . 2015. “Microalgae: Cultivation Techniques and Wastewater Phycoremediation.” Journal of Environmental Science and Health ‐ Part A Toxic/Hazardous Substances and Environmental Engineering 50, no. 6: 585–601. 10.1080/10934529.2015.994951. 25837561

[wer70276-bib-0048] Ruan, L. , M. Cheng , D. Xu , et al. 2025. “Nutrient Removal and Lipid Production Using Chlorella pyrenoidosa in Unsterilized Domestic Wastewater.” Waste and Biomass Valorization 16, no. 3: 1501–1509. 10.1007/s12649-024-02751-6.

[wer70276-bib-0049] Rumin, J. , E. Nicolau , R. Gonçalves de Oliveira Junior , C. Fuentes‐Grünewald , and L. Picot . 2020. “Analysis of Scientific Research Driving Microalgae Market Opportunities in Europe.” Marine Drugs 18, no. 5: 264. 10.3390/md18050264. 32443631 PMC7281102

[wer70276-bib-0050] Salbitani, G. , and S. Carfagna . 2021. “Ammonium Utilization in Microalgae: A Sustainable Method for Wastewater Treatment.” Sustainability 13, no. 2: 956. 10.3390/su13020956.

[wer70276-bib-0052] Santos, B. , F. Freitas , A. J. F. N. Sobral , and T. Encarnação . 2025. “Microalgae and circular economy: unlocking waste to resource pathways for sustainable development.” International Journal of Sustainable Engineering 18, no. 1: 2501488. 10.1080/19397038.2025.2501488.

[wer70276-bib-0053] Santos, F. M. , and J. C. M. Pires . 2018. “Nutrient Recovery From Wastewaters by Microalgae and Its Potential Application as Bio‐Char.” Bioresource Technology 267: 725–731. 10.1016/j.biortech.2018.07.119. 30082133

[wer70276-bib-0054] Sati, H. , M. Mitra , S. Mishra , and P. Baredar . 2019. “Microalgal Lipid Extraction Strategies for Biodiesel Production: A Review.” Algal Research 38: 101413. 10.1016/j.algal.2019.101413.

[wer70276-bib-0055] Shafiei‐Alavijeh, R. , M. Eppink , J. F. M. Denayer , E. Peeters , and K. Karimi . 2024. “Sustainable Biorefining of Chlorella vulgaris Into Protein, Lipid, Bioethanol, and Biogas With Substantial Socioeconomic Benefits.” Energy Conversion and Management 314: 118683. 10.1016/j.enconman.2024.118683.

[wer70276-bib-0056] Silva, S. , L. B. U. Melo , B. B. Borrego , L. H. Gracioso , E. A. Perpetuo , and C. A. O. do Nascimento . 2024. “Sugarcane Vinasse as Feedstock for Microalgae Cultivation: From Wastewater Treatment to Bioproducts Generation.” Brazilian Journal of Chemical Engineering 41: 911–921. 10.1007/s43153-023-00399-8.

[wer70276-bib-0057] Singh, V. , and V. Mishra . 2021. “Exploring the Effects of Different Combinations of Predictor Variables for the Treatment of Wastewater by Microalgae and Biomass Production.” Biochemical Engineering Journal 174: 108129. 10.1016/j.bej.2021.108129.

[wer70276-bib-0058] Solarte‐Toro, J. C. , and C. A. C. Alzate . 2021. “Biorefineries as the Base for Accomplishing the Sustainable Development Goals (SDGs) and the Transition to Bioeconomy: Technical Aspects, Challenges and Perspectives.” Bioresource Technology 340: 125626. 10.1016/j.biortech.2021.125626. 34325388

[wer70276-bib-0059] Soto, M. F. , C. A. Diaz , A. M. Zapata , and J. C. Higuita . 2021. “BOD and COD Removal in Vinasses From Sugarcane Alcoholic Distillation by Chlorella vulgaris: Environmental Evaluation.” Biochemical Engineering Journal 176: 108191. 10.1016/j.bej.2021.108191.

[wer70276-bib-0060] Souza, T. S. O. , and E. Foresti . 2013. “Sulfide‐Oxidizing Autotrophic Denitrification: An Evaluation for Nitrogen Removal From Anaerobically Pretreated Domestic Sewage.” Applied Biochemistry and Biotechnology 170, no. 5: 1094–1103. 10.1007/s12010-013-0261-8. 23640264

[wer70276-bib-0061] Standard Methods for the Examination of Water and Wastewater . 2025. “Method 4500‐NH_3_: Nitrogen (Ammonia).” https://www.edgeanalytical.com/wp‐content/uploads/Waste_SM4500‐NH3.pdf.

[wer70276-bib-0062] Stiles, W. A. V. , D. Styles , S. P. Chapman , et al. 2018. “Using Microalgae in the Circular Economy to Valorise Anaerobic Digestate: Challenges and Opportunities.” Bioresource Technology 267: 732–742. 10.1016/j.biortech.2018.07.100. 30076074

[wer70276-bib-0063] Su, Z. , M. Jalalah , S. A. Alsareii , et al. 2024. “Effect of Pharmaceutical and Domestic Wastewater Mixed Ratios on Microalgal Growth for Nutrients Removal Coupled With Biomass and Liquid Biofuel Generation.” Biomass Conversion and Biorefinery 14, no. 24: 32149–32161. 10.1007/s13399-023-04911-5.

[wer70276-bib-0064] Tran, D. T. , T. C. Van Do , Q. T. Nguyen , and T. G. Le . 2021. “Simultaneous Removal of Pollutants and High Value Biomaterials Production by Chlorella variabilis TH03 From Domestic Wastewater.” Clean Technologies and Environmental Policy 23, no. 1: 3–17. 10.1007/s10098-020-01810-5.

[wer70276-bib-0065] UNESCO . 2017. The United Nations World Water Development Report, 2017: Wastewater: The Untapped Resource. United Nations World Water Assessment Programme.

[wer70276-bib-0066] United Nations . 2025. Take Action for the Sustainable Development Goals. Sustainable Development Goals. https://www.un.org/sustainabledevelopment/sustainable‐development‐goals/.

[wer70276-bib-0067] Usui, N. , and M. Ikenouchi . 1997. “The Biological CO_2_ Fixation and Utilization Project by RITE(1)—Highly‐Effective Photobioreactor System—.” Energy Conversion and Management 38: S487–S492. 10.1016/S0196-8904(96)00315-9.

[wer70276-bib-0068] Vazirzadeh, A. , K. Jafarifard , A. Ajdari , and Y. Chisti . 2022. “Removal of nitRate and Phosphate From Simulated Agricultural Runoff Water by Chlorella vulgaris.” Science of the Total Environment 802: 149988. 10.1016/j.scitotenv.2021.149988. 34525699

[wer70276-bib-0069] Wang, Q. , X. Wang , Y. Hong , et al. 2022. “Microalgae Cultivation in Domestic Wastewater for Wastewater Treatment and High Value‐Added Production: Species Selection and Comparison.” Biochemical Engineering Journal 185: 108493. 10.1016/j.bej.2022.108493.

[wer70276-bib-0070] Wang, S. , Y. Mukhambet , S. Esakkimuthu , and A. E.‐F. Abomohra . 2022. “Integrated Microalgal Biorefinery—Routes, Energy, Economic and Environmental Perspectives.” Journal of Cleaner Production 348: 131245. 10.1016/j.jclepro.2022.131245.

[wer70276-bib-0071] Whitaker, M. , S. Rodrigues , G. Cooke , et al. 2025. “How COVID‐19 Affected Academic Publishing: A 3‐Year Study of 17 Million Research Papers.” International Journal of Epidemiology 54, no. 3: dyaf058. 10.1093/ije/dyaf058. 40421615 PMC12107239

[wer70276-bib-0072] Xin, L. , H. Hong‐ying , G. Ke , and S. Ying‐xue . 2010. “Effects of Different Nitrogen and Phosphorus Concentrations on the Growth, Nutrient Uptake, and Lipid Accumulation of a Freshwater Microalga Scenedesmus sp.” Bioresource Technology 101, no. 14: 5494–5500. 10.1016/j.biortech.2010.02.016. 20202827

[wer70276-bib-0073] Yin, H. , T. Huang , B. Wang , et al. 2025. “Dissolved Organic Matter Sources in Urban Outfall Water Before and During Rainfall, Indicated via Its Fluorescence Fingerprinting.” Applied Geochemistry 189: 106447. 10.1016/j.apgeochem.2025.106447.

[wer70276-bib-0074] Yu, S. , Z. Chen , M. Li , S. Qiu , Z. Lv , and S. Ge . 2024. “Principles, Challenges, and Optimization of Indigenous Microalgae‐Bacteria Consortium for Sustainable Swine Wastewater Treatment.” Bioresource Technology 406: 131055. 10.1016/j.biortech.2024.131055. 38944316

[wer70276-bib-0075] Zabochnicka, M. , M. Krzywonos , Z. Romanowska‐Duda , S. Szufa , A. Darkalt , and M. Mubashar . 2022. “Algal Biomass Utilization Toward Circular Economy.” Life 12, no. 10: 1480. 10.3390/life12101480. 36294915 PMC9605372

[wer70276-bib-0076] Zhao, J. , L. Peng , and X. Ma . 2025. “Innovative Microalgae Technologies for Mariculture Wastewater Treatment: Single and Combined Microalgae Treatment Mechanisms, Challenges and Future Prospects.” Environmental Research 266: 120560. 10.1016/j.envres.2024.120560. 39647683

[wer70276-bib-0077] Zhao, L.‐S. , K. Li , Q.‐M. Wang , et al. 2017. “Nitrogen Starvation Impacts the Photosynthetic Performance of Porphyridium cruentum as Revealed by Chlorophyll a Fluorescence.” Scientific Reports 7, no. 1: 8542. 10.1038/s41598-017-08428-6. 28819147 PMC5561210

[wer70276-bib-0078] Zhao, X. , J. Yang , R. Han , et al. 2025. “Modular Constructed Wetlands for Treatment of Rural Domestic Wastewater: Laboratory Performance and Field Application.” Sustainability 17, no. 10: 4427. 10.3390/su17104427.

[wer70276-bib-0079] Zhu, S. , R. Jiang , L. Qin , et al. 2022. “Integrated Strategies for Robust Growth of Chlorella vulgaris on Undiluted Dairy Farm Liquid Digestate and Pollutant Removal.” Science of the Total Environment 852: 158518. 10.1016/j.scitotenv.2022.158518. 36063926

